# Pathogenic fungus *Ustilago maydis* exploits the lateral root regulators to induce pluripotency in maize shoots

**DOI:** 10.1111/nph.70843

**Published:** 2025-12-26

**Authors:** Mamoona Khan, Nithya Nagarajan, Kathrin Schneewolf, Caroline Marcon, Danning Wang, Frank Hochholdinger, Peng Yu, Armin Djamei

**Affiliations:** ^1^ Department of Plant Pathology Institute of Crop Science and Resource Conservation (INRES), University of Bonn Nussallee 9 53115 Bonn Germany; ^2^ Leibniz Institute of Plant Genetics and Crop Plant Research (IPK) OT Gatersleben, Corrensstraße 3 D‐06466 Stadt Seeland Germany; ^3^ Crop Functional Genomics, Institute of Crop Sciences and Resource Conservation, University of Bonn 53113 Bonn Germany; ^4^ Plant Genetics, TUM School of Life Sciences, Technical University of Munich 85354 Freising Germany; ^5^ Plant Breeding, TUM School of Life Sciences, Technical University of Munich 85354 Freising Germany

**Keywords:** ARF19, ARF7, gall, lateral root formation, LOB‐domain protein, Tip effector, Topless corepressor, *Ustilago maydis*

## Abstract

- Biotrophic plant–pathogens secrete effector molecules to redirect and exploit endogenous signaling and developmental pathways in their favor. The biotrophic fungus *Ustilago maydis* causes galls on all aerial parts of maize. However, the responsible gall‐inducing effectors and corresponding plant signaling pathway(s) remain largely unknown.
- Using molecular and genetic approaches, and transcriptomic comparisons in maize, we identify downstream targets and developmental consequences of the plant TOPLESS (TPL)‐interacting protein (Tip) effectors in gall formation.
- We demonstrate that Tip4 derepress *AtARF7/AtARF19* branch of auxin signaling, leading to the formation of pluripotent calli without the external addition of phytohormones. Comparative transcriptomics in maize further reveals a significant overlap of genes upregulated during *U. maydis*‐triggered leaf gall formation and the developmental initiation of lateral roots (LRs). Additionally, we show that this process involves the transcriptional upregulation of downstream LATERAL ORGAN BOUNDARIES DOMAIN (LBD) transcription factors. Homozygous mutations in two LBD genes (*ra2*, *rtcs*) resulted in significantly reduced gall formation in maize.
- Taken together, our results suggest that *U. maydis* hijacks the LR initiation pathway to trigger gall formation in maize shoots, revealing key effectors and host pathways exploited by biotrophic pathogens.

## Introduction

Pathogen‐induced plant galls are the morphological outcome of abnormal growth of plant tissue induced by the manipulative activities of the invading organism. These result from the increased proliferation (hyperplasia) and/or increased cell size of a group of cells (hypertrophy). Although more than a century of research, neither the physiological networks nor the exact mechanisms of gall induction and development have been fully elucidated (Dodueva *et al*., 2020). *Ustilago maydis*, a basidiomycete fungus, causes common smut disease in maize, infecting all aerial parts of the host, including the stem, leaves, and flowers. Early infection symptoms include local chlorosis and anthocyanin accumulation. A hallmark of *U. maydis* infection in maize is the formation of galls on aboveground organs, which act as sink tissues that, by the end of the fungal proliferation cycle, become filled with countless black diploid teliospores observable as smut symptoms (Brefort *et al*., 2009). *Ustilago maydis*induced gall formation results from intensive cell division and expansion in specific cell types (Matei *et al*., 2018). Notably, not all cells in the infected tissue proliferate, indicating that this process is cell‐type or zone‐specific. For instance, in maize flowers, *U. maydis* colonizes only immature, undifferentiated anther cells with meristematic activity, sustaining cell division beyond normal development (Gao *et al*., 2013; Lin *et al*., 2021). In leaf galls, differentiated bundle sheath cells resume cell divisions, and mesophyll cells enlarge (Matei *et al*., 2018). However, the molecular mechanisms involved in gall formation remain poorly understood.

The *U. maydis* genome encodes 467 predicted secreted proteins, and several show distinct expression patterns during maize colonization (Lanver *et al*., 2018); however, only a few have been functionally characterized. Most known effectors are linked to immune suppression (Doehlemann *et al*., 2009; Navarrete *et al*., 2021, 2022; Saado *et al*., 2022), host metabolism, and phytohormone manipulation (Reineke *et al*., 2008; Djamei *et al*., 2011; Tanaka *et al*., 2014; Rabe *et al*., 2016; Ma *et al*., 2018) or fungal development in the host (Tanaka *et al*., 2020; Lin *et al*., 2023; Weiland *et al*., 2023). Yet little is known about effectors directly involved in gall formation. For example, See1 reactivates DNA synthesis in leaf galls but not in tassel galls; its deletion inhibits hyperplastic cell division (Redkar *et al*., 2015). Sts2 promotes hyperplasia by transcriptional dysregulation, and its deletion reduces cell division in bundle sheath cells (Zuo *et al*., 2023). Another effector, ApB73, plays a cultivar‐specific role in gall formation (Stirnberg & Djamei, 2016). The persistence of galls despite the deletion of these effectors implies functional redundancy or cooperation among multiple pathways in this complex developmental process.

Auxin, a central regulator of plant growth, also plays a role in plant–pathogen interactions (Kunkel & Johnson, 2021; Nagarajan *et al*., 2023). During *U. maydis* infection, auxin levels increase, partly due to fungal INDOLE‐3‐ACETIC ACID (IAA) production, although this alone is not essential for gall formation (Reineke *et al*., 2008). Auxin‐responsive genes are upregulated in infected tissues, and at least 10 *U. maydis* effectors (Jsi1, Nkd1, and Tip1–8) target TOPLESS (TPL) transcriptional corepressors to modulate auxin signaling (Darino *et al*., 2021; Bindics *et al*., 2022; Navarrete *et al*., 2022; Huang *et al*., 2023; Khan *et al*., 2024). A pentuple deletion mutant of these (*Δtips1‐5*) led to a significant reduction in gall sizes and numbers (Bindics *et al*., 2022), highlighting the crucial role of TPL proteins in the biotrophic stage of *U. maydis*. However, the specific downstream targets of TPL‐controlled signaling remain to be explored. TPL corepressors in auxin signaling are recruited by AUXIN (Aux)/IAA proteins through conserved Ethylene‐responsive element binding factor‐associated Amphiphilic Repression (EAR) motifs (Tiwari *et al*., 2004; Szemenyei *et al*., 2008). Aux/IAAs do not bind DNA directly but interact with AUXIN RESPONSE FACTORs (ARFs) to suppress transcription. Since the ability of Aux/IAAs to modulate transcription is dependent on ARFs, the presence of different ARF complements in different cells can also affect auxin signaling specificity (Rademacher *et al*., 2012; Bargmann *et al*., 2013; Leyser, 2018).

Auxin also regulates pluripotency and organogenesis by regulating fate, division, and differentiation, enabling new organ formation throughout the plant lifecycle (Perianez‐Rodriguez *et al*., 2014). Lateral root (LR) formation in plants is an example of postembryonic development. In *Arabidopsis thaliana*, LRs originate exclusively from pericycle founder cells in response to local auxin maxima (Celenza Jr. *et al*., 1995; Casimiro *et al*., 2001), which results in the derepression of AtARF7 and AtARF19, and the expression of *LATERAL ORGAN BOUNDARY* (*LOB*) *DOMAIN* (*LBD*) transcription factors (TFs; Wilmoth *et al*., 2005; Okushima *et al*., 2007; Lee *et al*., 2009). Formation of callus, proliferating masses of pluripotent cells from various differentiated explants, is often the first step in *in vitro* plant generation and is also initiated by elevated auxin levels in the tissue culture medium (Lardon & Geelen, 2020). Intriguingly, callus formation involves an ectopic activation of the root primordia development program from pericycle or pericycle‐like cells, even when derived from aerial organs, such as cotyledons and petals (Atta *et al*., 2009; Sugimoto *et al*., 2010; Xu *et al*., 2012). Auxin accumulation in the founder cells mediates the degradation of IAA14/SOLITARY ROOT, which releases AtARF7 and AtARF19 that in turn upregulate the expression of *AtLBD16*, *AtLBD18*, and *AtLBD29* during callus formation (Lardon & Geelen, 2020). Ectopic LBD expression can induce callus without exogenous hormones, while their suppression inhibits auxin‐induced callus formation (Okushima *et al*., 2005; Fan *et al*., 2012). The LBD family comprises 43 members in *A. thaliana* (Fan *et al*., 2012) and 49 in *Zea mays* (Zhang *et al*., 2020); however, only a few have been shown to regulate LR organogenesis and callus formation in *A. thaliana* (Feng *et al*., 2012; Goh *et al*., 2012; Lee *et al*., 2013; Pandey *et al*., 2018), and functional knowledge on LBDs in maize is limited. *Rootless concerning crown and seminal roots (rtcs)* and *rtcs‐like (rtcl)* are two paralogous genes in maize and orthologues of *A. thaliana AtLBD29* (Berardini *et al*., 2015) that control seminal and postembryonic shoot‐borne root formation (Taramino *et al*., 2007; Xu *et al*., 2015). *ramosa 2* (*ra2*) is an orthologue of *A. thaliana AtLBD25* and *AtLOB* genes (Zhang *et al*., 2020) and controls inflorescence branching (Bortiri *et al*., 2006). Although *ra2* is highest expressed in the roots of maize (Winter *et al*., 2007; Hoopes *et al*., 2019; Woodhouse *et al*., 2021), its direct role in LR formation in maize has not been explored.

In this study, we reveal the role of a set of Tip effectors in *U. maydis‐induced* cellular dedifferentiation, cell division, and gall formation. We show that a single Tip effector can induce cellular dedifferentiation leading to callus formation and cell division in transgenic *A. thaliana* plants. Furthermore, we provide genetic evidence that this process relies on the expression of *AtARF7* and *AtARF19* TFs. It is also demonstrated that *U. maydis* induces the expression of *Zmarf27*, an orthologue of *AtARF7* and *AtARF19*, and the LBDs, which are dominant susceptibility factors in maize during biotrophic colonization. Transcriptomic overlap between gall formation and LR development supports the notion that *U. maydis* exploits parts of the LR formation pathway in maize for leaf‐gall induction. Ultimately specific maize LBD mutants (ra2 and rtcs) show significantly reduced gall formation in comparison to wild type maize upon *U. maydis* infection.

## Materials and Methods

### Molecular cloning

Cloning was performed using either Greengate (Lampropoulos *et al*., 2013) or Golden Gate (Katzen, 2007) cloning systems as described previously (Khan *et al*., 2024). Mach1 competent cells (Thermo Fisher Scientific, Waltham, MS, USA) were used for all DNA manipulations and were grown in a double Yeast Extract Tryptone medium (dYT) liquid medium or on YT agar plates with the required antibiotic supplements. *pXVE‐HA‐mCherry‐(effector lacking signal peptide)* was the construct used for generating transgenic effector lines.

### Plant material and growth conditions

*Arabidopsis thaliana* (L.) Heynh. Columbia was a wild‐type used for generating all transgenic lines. *pAtLBD16:GUS* reporter line and T‐DNA insertional mutant *arf7*, *arf19* lines were obtained from the Nottingham Arabidopsis Stock Centre (NASC) under the ID N68141 and N24629 respectively and *ra2‐R* maize mutant seeds were received from maize genetic and cooperation stock center (USA). *A. thaliana* for dipping were grown in controlled short‐day conditions (8 h : 16 h, 21°C : 2°C, light : dark) while maize was grown in distinct conditions (14 h : 10 h, 28°C : 20°C, light : dark). Floral dipping was used to generate two transgenic *A. thaliana* lines for each plasmid construct with similar protein levels, selected with glufosinate‐ammonium on soil. For phenotyping, the plants were grown on solidified half‐strength MS agar media, supplemented with 1% (w/v) sucrose plates in growth cabinets (Panasonic environmental test chamber, Type: MLR‐352H‐PE) at 21°C ± 2°C and on long days of 16 h : 8 h, light : dark cycles with 80 μmol/m^2^/s intensity. For estradiol treatments, 7‐d‐old plate‐grown seedlings were transferred either to dimethyl sulfoxide (DMSO) or 10 μM β‐estradiol containing ½ MS plates, and pictures were taken 5, 10, 15, and 20 d after transfer to the plates or otherwise stated. Experiments were repeated at least three times.

### Maize infection assays

Maize (*Zea mays* L.) variety Early Golden Bantam, Old Seeds, Madison, WI, USA, was used for all infections until otherwise stated. *Ustilago maydis* (DC.) Corda progenitor strain SG200 was used to infect seven‐day‐old maize seedlings for quantitative reverse transcription polymerase chain reaction (qRT‐PCRs) and nine‐day‐old maize seedlings for mutant analyses as described in detail previously (Redkar & Doehlemann, 2016). For qRT‐PCRs, leaf samples were collected *c*. 1 cm below the hole of the syringe in infected leaves 4 days post infection (dpi), whereas symptom scoring was performed at 12 dpi according to Kamper *et al*. (2006). Symptom scores were assessed using the Fisher's exact test in R, as described previously (Stirnberg & Djamei, 2016).

### Confocal microscopy

Confocal microscopy was performed with a Leica SP8 confocal microscope. mCherry was excited at 561 nm, and emission was collected between 578 and 648 nm. Images were processed using the LAS‐X software from Leica.

### qRT‐PCR and comparative transcriptome analyses

mRNA was extracted from the ground powder of leaves using a New England Biolabs GmbH (Frankfurt am Main, Germany) RNA extraction kit, and cDNA was synthesized using a Thermo Scientific RevertAid First Strand cDNA Synthesis Kit. Quantitative polymerase chain reaction (qPCRs) were performed with GoTaq qPCR mix (NEB, cat. no. A6001) according to the manufacturer's instructions. Relative amounts of amplicons were calculated according to the 2^−ΔΔCt^ method (Livak & Schmittgen, 2001). The results are the average of four biological replicates. *Zmcdk* (Zm00001eb350890) was used as housekeeping gene (Lin *et al*., 2014).

### Biostatistical analysis of lateral root dataset and *U. maydis* dataset

To understand the biological pathway during LR initiation and callus formation in maize, a previously published transcriptomic dataset of *U. maydis*‐infected maize leaf tissues (Lanver *et al*., 2018), designated here as *U. maydis‐*maize experiment, was downloaded, and only the time point 4 dpi was selected, as it is the time when galls are formed. The LR transcriptomics, which used cell‐type‐specific RNA sequencing of LR mutant vs wild‐type maize plants via laser capture microdissection, designated here as the *LR* experiment, was generated in Baer *et al*. (2025) and is available under sequence read archive accession no. PRJNA1366082. Genes preferentially expressed in phloem‐pole pericycle cells were defined as ‘LR‐enriched’, while genes expressed in xylem‐pole pericycle cells were defined as ‘LR‐depleted’. DESeq2 was used to test for gene expression changes. For both datasets, only genes with at least five normalized read counts for at least one time point/cell type from three replicates were considered as expressed. A differential expression threshold of log_2_ fold change > 1 and Benjamini–Hochberg‐adjusted *P* value <0.05 was used in both datasets. The overlapping and differentially expressed genes between datasets were visualized by Venn diagram (https://bioinformatics.psb.ugent.be). A chi‐squared (χ^2^) test in R (v.1.4.1717) was used to determine whether the two transcriptome datasets share a significant correlation. The overlapping genes between datasets were functionally characterized by Gene Ontology (GO) annotation and enrichment analysis via AgriGO (v.2, http://systemsbiology.cpolar.cn/agriGOv2/).

## Results

### Induction of Tip expression leads to strong morphological phenotypes *in planta*

**Fig. 1:**
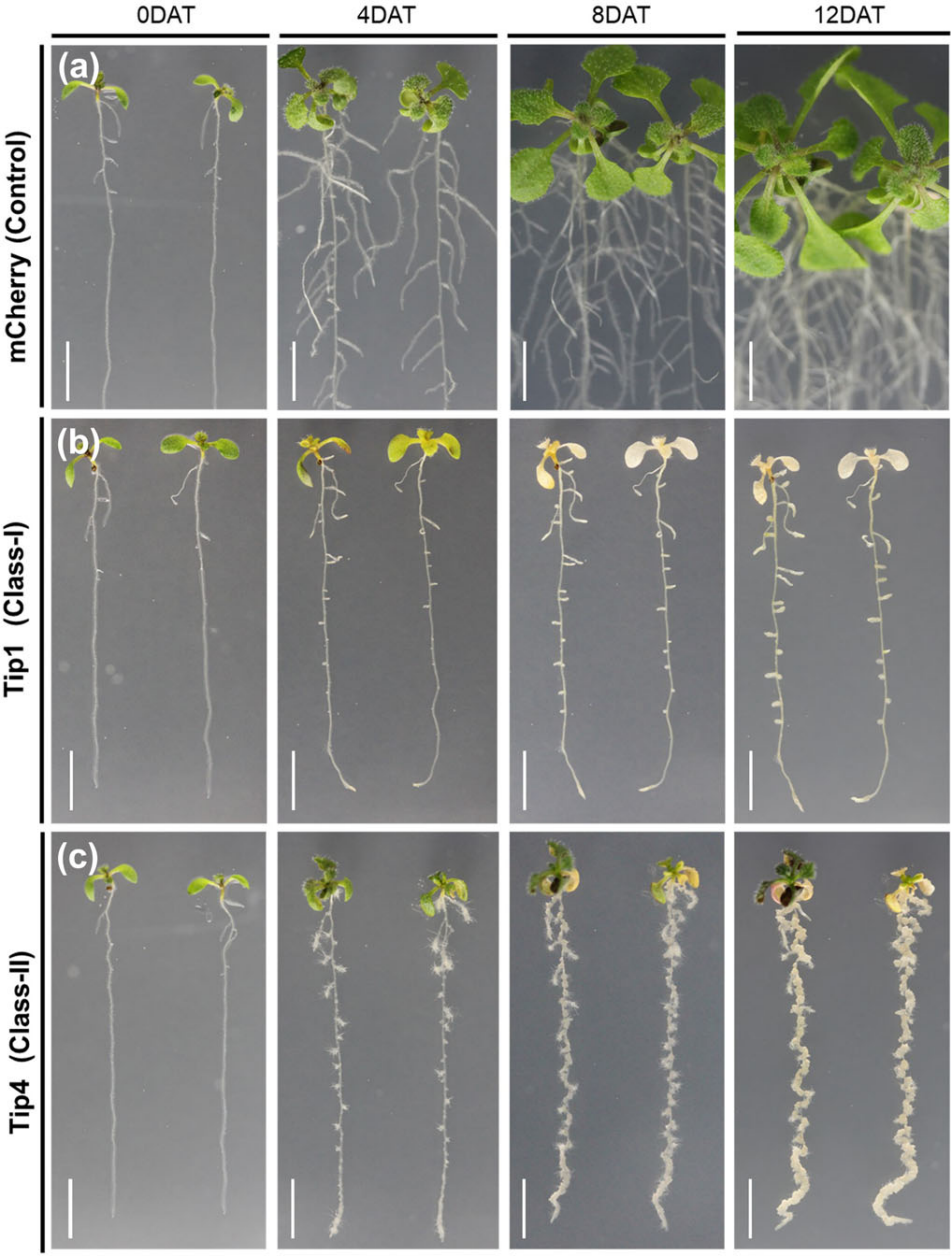
Expression of TOPLESS‐interacting protein (Tip) effectors induces strong morphological phenotypes in *Arabidopsis thaliana*. Seven‐day‐old *A. thaliana* seedlings expressing (a) *pXVE: HA‐mCherry*, (b) *pXVE: HA‐mCherry‐Tip1*, or (c) *pXVE: HA‐mCherry‐Tip4* were transferred to agar plates containing estradiol, and images were taken at 0, 4, 8, and 12 d after transfer. DAT, d after transfer. Bars, 1 cm.

**Fig. 2:**
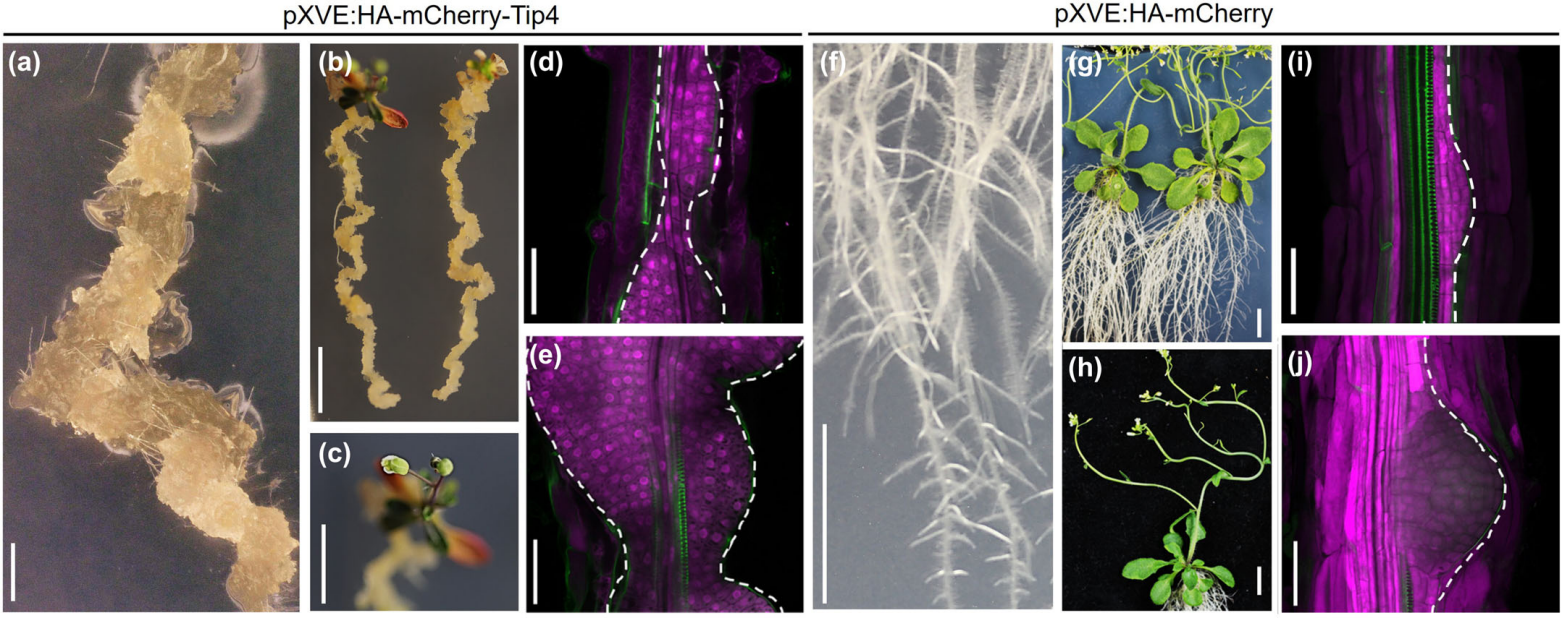
TOPLESS‐interacting protein (Tip) effector 4 induces callus‐like structures along the primary root. (a, b) Images of plants expressing *pXVE:HA‐mCherry‐Tip4* at 12 d after transfer (DAT) (a) and 29 DAT (b) to estradiol induction medium, showing extensive callus formation along the primary root. (c) Flowering observed in a *pXVE:HA‐mCherry‐Tip4*‐expressing plant at 29 DAT. (d, e) Confocal images of the root elongation zone in plants expressing *pXVE:HA‐mCherry‐Tip4* showing morphologies associated with callus formation. (f, g) Images of control plants expressing *pXVE:HA‐mCherry* at 12 DAT (f) and 29 DAT (g) to estradiol induction medium, showing normal root development. (h) Flowering observed in a *pXVE:HA‐mCherry*‐expressing plant at 29 DAT. (i, j) Confocal images of the root elongation zone in control plants expressing *pXVE:HA‐mCherry* showing morphologies associated with lateral root formation. Green fluorescence indicates auramine staining of the root vasculature. Bars, a = 1 mm; b, c, f, g, h = 1 cm; d, e, i, j = 50 μm.

Although *U. maydis* has at least 10 TPL‐interacting effectors (Jsi1, Nkd1, Tip1‐8) (Darino *et al*., 2021; Bindics *et al*., 2022; Navarrete *et al*., 2022; Huang *et al*., 2023; Khan *et al*., 2024), it is not clear why so many are needed and which downstream pathways are activated. As TPL corepressors are central, conserved negative transcriptional regulators in all land plants, we generated transgenic *A. thaliana* lines expressing each of these Tip effectors to study their individual biological activity. Intriguingly, this resulted in a range of strong morphological phenotypes across two independent transgenic lines tested compared to an mCherry‐expressing control (Supporting Information Figs 1a, S1–S3). More specifically, the induction of *Tip1*, *Tip2*, *Tip8*, *Jsi1*, and *Nkd1* expression in *A. thaliana* led to chlorophyll (Chl) loss in the cotyledons and leaves, and overall growth arrest already clearly visible at 4 d after transfer (Figs 1b, S1, S2), while expression of *Tip3*, *Tip4*, *Tip5*, *Tip6*, and *Tip7* led to a strong induction of LR formation and inhibition of primary root length (Figs 1c, S3, S4). Noticeably, the TPL‐interacting effectors that lead to overall growth inhibition and complete Chl loss in *A. thaliana* previously showed a cell death phenotype upon transient overexpression in *Nicotiana benthamiana* leaves (Darino *et al*., 2021; Navarrete *et al*., 2022; Khan *et al*., 2024). Based on their cell death‐inducing feature vs LR induction abilities, we categorized the 10 known TPL‐interacting effectors into two classes (Table S1), class I and II represented by Tip1 and Tip4, which exhibited the strongest phenotype of their respective class (Fig. 1b,c). Strikingly, in the strongest case of class II effectors (Tip3, Tip4, and Tip6), the LRs did not grow in size; instead, the whole root thickened due to the initiation of undifferentiated (callus‐like) structures that formed without the external addition of any phytohormones on the plate (Figs 1c, 2a–e, S3A,B,D) as compared to control plants (Figs 1a, 2f–j, S1A). Surprisingly, this phenotype was restricted to the roots only; in the shoots, the growth either slowed down or the leaves turned pale. These plants continued to survive, and the shoots even flowered (Fig. 2c). To test whether these undifferentiated (callus‐like) structures are indeed pluripotent, we transferred them (without their shoots) to the shoot induction media used for *A. thaliana* regeneration. This led to greening and shoot formation (Fig. S5), supporting this conclusion. To summarize, ectopic expression of TPL‐interacting effectors in *A. thaliana* resulted in extreme morphological phenotypes, which could be grouped into two distinct classes. In this study, we focus on class II Tip effectors whose expression leads to LR and callus formation in *A. thaliana* for in‐depth investigation.

### Overexpression of class II Tips leads to the formation of calli in *A. thaliana* roots through the TPL‐AtARF7/AtARF19‐AtLBD16 pathway

**Fig. 3:**
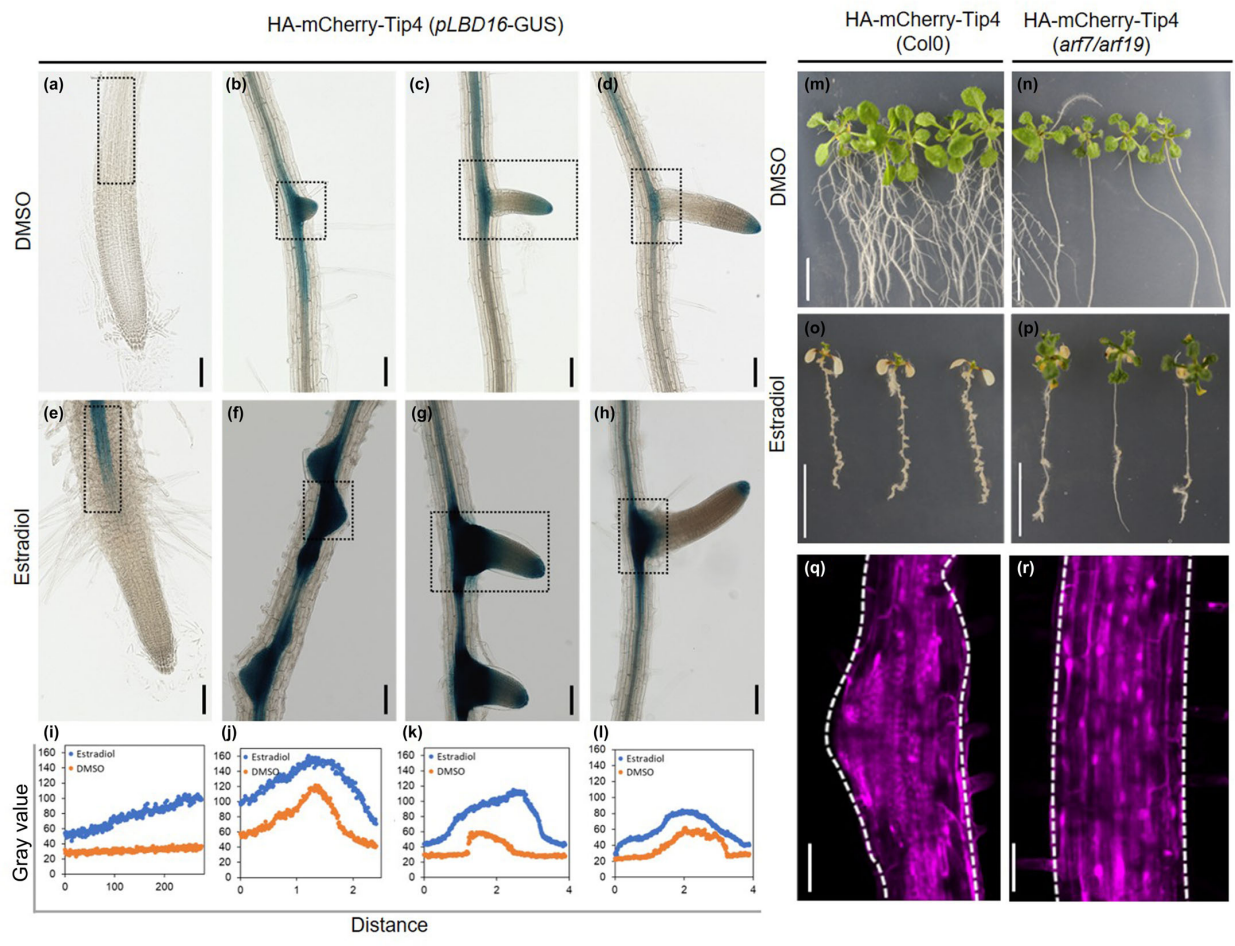
TOPLESS‐interacting protein (Tip) 4 effector‐induced root callus formation requires AtARF7 and AtARF19‐mediated *AtLBD16* expression. (a–h) Digital microscopy images of seedlings expressing *pLBD16:GUS* in a *pXVE:HA‐mCherry‐Tip4* background. Seven‐day‐old seedlings grown on ½ MS agar were transferred to either dimethyl sulfoxide (DMSO)‐containing medium (a–d) or 10 μM estradiol (e–h) for 4 d before β‐glucuronidase (GUS) staining. Bars, 100 μm. (i ‐l) The GUS staining intensities were evaluated from the images (a–h) in the indicated regions (rectangles) by using fiji (Schindelin *et al*., 2012) as described previously (Beziat *et al*., 2017). Orange line in each graph indicate GUS intensity in DMSO‐treated seedling and the blue line in each graph represents GUS intensity in estradiol ‐treated seedling. (m–p) Phenotypes of seedlings expressing *pXVE:HA‐mCherry‐Tip4* in wild‐type Columbia (Col‐0; m, o) or *arf7/arf19* mutant background (n, p) 10 d after transfer to plates containing DMSO (m, n) or 10 μM estradiol (o, p). Bars, = 1 cm. (q, r) Confocal microscopy of 11‐d‐old *Arabidopsis thaliana* roots expressing *pXVE:HA‐mCherry‐Tip4* in Col‐0 (q) or *arf7/arf19* background (r), showing Tip4 effector protein expression 4 d after induction. Bars, 50 μm.

Expression of class II *U. maydis* Tip effectors (Tip3, Tip4, Tip5, Tip6, and Tip7) led to the initiation of LRs and pluripotent callus in the *A. thaliana* roots. Similar phenotypes have been described previously for LBD TF overexpression in *A. thaliana*, which act downstream of *AtARF7* and *AtARF19* during LR formation and for callus formation (Fan *et al*., 2012; Liu *et al*., 2018). We therefore hypothesized that class II Tips, following their specific interaction with the TPL class of corepressors (Bindics *et al*., 2022; Navarrete *et al*., 2022), interfere with the binding of Aux/IAA corepressors and derepress the auxin signaling cascade associated with LR and callus formation in *A. thaliana*. To test this, we first crossed *pAtLBD16:GUS* (the expression of β‐glucuronidase (GUS) reporter is driven under the control of the LBD16 promoter; Bargmann *et al*., 2014) with the Tip4 expression line *pXVE:HA‐mCherry‐Tip4* (Tip4 representative for the class II phenotypes) and observed the GUS‐reporter expression pattern. Microscopic observation revealed that *pAtLBD16:GUS* expression was strongly induced in a Tip4‐dependent manner in seedling roots after estradiol induction (Figs 3a–l, S6). This implies that Tip‐targeted TPLs are major negative regulators of *AtLBD16* in the root, but there may be other factors in *Arabidopsis* shoots.

To provide further genetic evidence that morphological phenotypes of class II overexpressing effector lines are a result of the derepression of a branch of the auxin signal cascade that controls LR and callus formation through *AtARF7* and *AtARF19*, we examined the overexpression phenotypes of Tip4 in an *arf7/arf19* T‐DNA insertional double mutant background (Okushima *et al*., 2005), which does not produce LRs. For this purpose, we crossed *pXVE:HA‐mCherry‐Tip4* (Fig. 3m) expressing *A. thaliana* plants with the *arf7/arf19* double mutant (Fig. 3n) and observed the phenotypes of homozygous *pXVE:HA‐mCherry‐Tip4 arf7/arf19* seedlings in the F2 generation after DMSO (Fig. 3m,n) or estradiol (Fig. 3o–p) treatments. As shown in Fig. 3m–r, whereas the Tip4‐expression‐mediated root length inhibition stayed unaffected (Fig. S4), the Tip4‐expression‐mediated induced callus formation was largely abolished in the *arf7/arf19* background (Fig. 3o,p). Furthermore, the effect on leaf chlorosis upon class II Tip expression is strongly reduced in the *arf7/arf19* mutant background (Fig. 3o,p). These results suggest a direct role of *AtARF7* and *AtARF19* in both phenomena, the Tip effector‐mediated callus formation in *A. thaliana* via the LR pathway, as well as the observed effects of Chl loss in the leaf chloroplasts.

### *Ustilago maydis* induces the expression of genes involved in lateral root formation during biotrophy in maize leaves

**Fig. 4:**
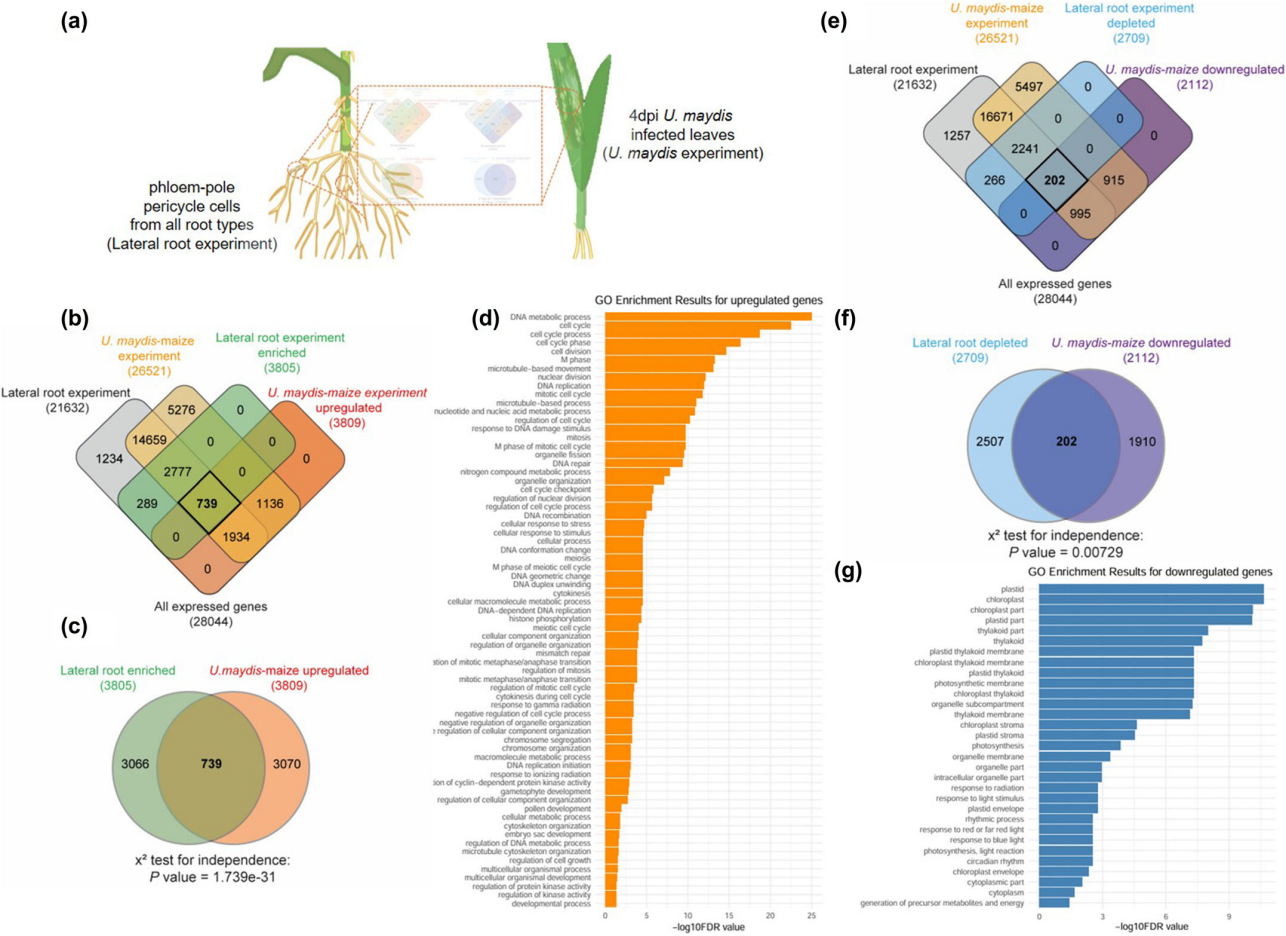
*Ustilago maydis* induces the expression of central regulators of the lateral root programming in maize shoots (a) Schematic overview of the experimental design. Transcriptomic data from *U. maydis‐*infected maize leaf tissue at 4 d post‐infection (4 dpi), the stage of gall formation (designated here as *U. maydis‐*maize experiment), were compared with gene expression profiles from phloem‐pole pericycle cells, the cell type responsible for LR initiation in maize, of 6‐wk‐old plants (designated here as lateral root (LR) experiment). (b) 4‐way Venn diagrams illustrating the overlap pattern of four different transcriptional datasets: expressed genes of LR experiment (21632), expressed genes of *U. maydis* maize infection experiment (26521), LR initiation enriched genes (3805), and *U. maydis* maize infection upregulated genes (3809) at 4 dpi. (c) Commonly upregulated genes between transcripts enriched in LR initiation cells and transcripts upregulated in 4dpi *U. maydis*‐infected maize. (d) The top enriched Gene Ontology (GO) terms are shown for biological processes. Bar thickness represents the number of associated genes, while bar height indicates significance (−log_10_ FDR). Terms with false discovery rate (FDR) < 0.05 were considered significantly enriched. (e) 4‐way Venn diagrams illustrating the overlap pattern of four different transcriptional datasets: expressed genes of LR experiment (21632), expressed genes of *U. maydis* ‐ maize infection experiment (26521), LR initiation depleted genes (2709), and *U. maydis ‐* maize infection downregulated genes (2112) at 4 dpi. (f) Commonly downregulated genes between transcripts depleted in LR initiation cells and transcripts downregulated at 4 dpi *U. maydis* maize infection. Statistical significance was assessed using the chi‐squared test for independence. (g) The top enriched GO terms are shown for biological processes. Bar thickness represents the number of associated genes, while bar height indicates significance (−log_10_ FDR). Terms with FDR < 0.05 were considered significantly enriched.

To estimate the role of the LR and callus formation pathway during *U. maydis* biotrophy on maize at the transcriptomic level, we compared previously published transcriptomic data of *U. maydis*‐infected maize leaf tissues at 4 dpi, the time point of gall formation (Lanver *et al*., 2018), with transcripts differentially regulated in phloem‐pole pericycle cells, the cell type, which gives rise to LRs in maize (Fig. 4a; Yu *et al*., 2016). A comparison of these two datasets indicates several genes are commonly regulated during these two biological processes (Fig. 4). In total, 28 044 genes were expressed in sum of the two independent experiments (LR experiment and *U. maydis*‐maize infection experiment). Among 20 109 commonly expressed genes, we found 3805 genes enriched in phloem‐pole pericycle cells, whereas upon *U. maydis* infection, 3809 maize genes are significantly (fold change > 2; FDR < 0.05) induced at 4 dpi (Fig. 4b). Between the two datasets of upregulated genes, 739 genes are upregulated in common, which is highly significant in a chi‐squared test for independence (*P* = 1.739e‐31; Fig. 4c).

Subsequently, the 739 commonly induced genes between the datasets were functionally classified according to GO terms using agriGOv2. In total, 69 GO terms belonging to different biological processes displayed significant overrepresentation (false discovery rate (FDR) < 0.05) (Dataset S2; Fig. 4d). Of all GO terms, the most enriched are involved in processes of cell cycle and cell division, suggesting the potential linkage of LR initiation and U. maydis‐induced gall formation in leaves. Also, the overlap of 202 commonly downregulated genes (Fig. 4e) between the *U. maydis*‐maize infection data and the LR initiation shows high significance in a chi‐squared test for independence (*P* = 0.00729; Fig. 4f). Analysis of the 202 genes (Fig. 4e,f) from 2709 genes transcriptionally underrepresented in phloem‐pole pericycle cells showed enrichment for 31 GOs (FDR < 0.05; Dataset S3; Fig. 4g). In particular, these involved photosynthesis and chloroplast‐related pathways, consistent with sink‐tissue formation and loss of photosynthetic activity at the place of gall induction by *U. maydis*.

Taken together, comparison of the two independent transcriptomic datasets generated from very different tissues and with different biological questions shows a significant overrepresentation of commonly up‐ and downregulated genes. This supports the notion that the shoot‐infecting fungus *U. maydis* recruits the LR pathway during the induction of galls on maize shoots.

### *Ustilago maydis* induces the expression of maize *Zmarf27*, the orthologue of *AtARF7* and *AtARF19* , in maize leaves

**Fig. 5:**
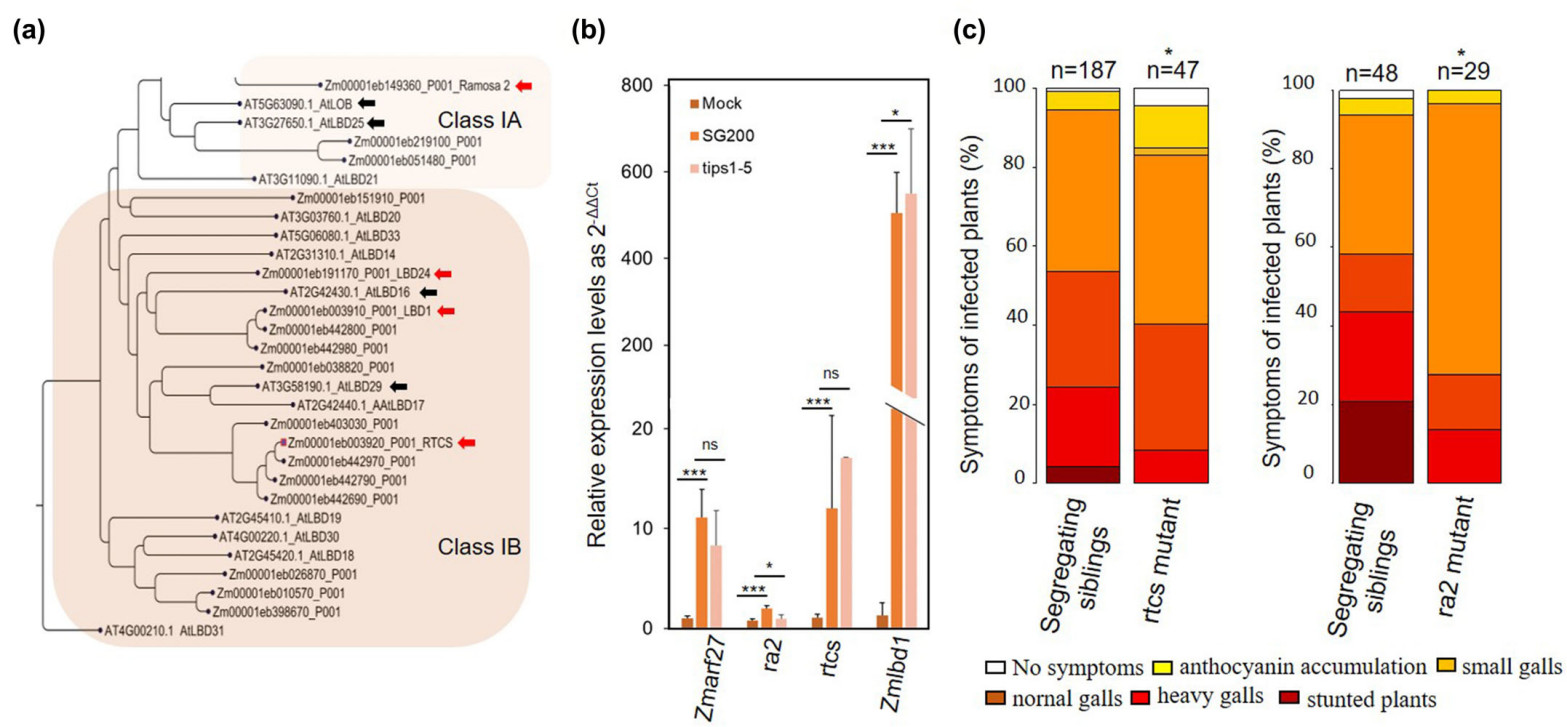
*Zmarf27* and certain ZmLBDs (*ra2*, *rtcs*, *Zmlbd1*) are required for *Ustilago maydis‐induced* gall formation. (a) The phylogeny of *LATERAL ORGAN BOUNDARY* (LOB) domains (LBD) proteins of class‐IA and class IB of *Arabidopsis thaliana* and *Zea mays*. The phylogenetic tree was reconstructed using aligned amino acid sequences of LOB domain proteins as an unrooted tree using CLC‐Genomics software 20.04 and a bootstrap value of 1000. Red arrows point to maize genes studied in the b,c and black arrows point to their *A. thaliana* orthologues. (b) The expression levels of *Zmarf27*, *ra2*, *rtcs*, and *Zmlbd1* were tested by quantitative real‐time PCR analyses at 4 d post‐infection of *U. maydis* SG200 solopathogenic strain, *Δtips1‐5* mutant strain or mock (water)‐infected maize seedlings. The 2^−ΔΔCt^ values were calculated and plotted as relative expression levels compared with mock‐infected plants as negative control. Error bars indicate SD. Significant differences between mock control and SG200‐infected leaves, and *Δtips1‐5* mutant strain were analyzed by Student's t‐test analysis (*, *P* < 0.05; ***, *P* < 0.005, ns, not significant). Data represent an average of four biological replicates. (c) Nine‐day‐old recessive homozygous maize mutants in *rtcs* or *ra2* genes were infected with *U. maydis* solopathogenic strain SG200 and compared to segregating population controls. Significant differences were analyzed by Fisher's exact test (*, *P* < 0.05).

Next, we hypothesized that class II Tip effectors induce gall formation in maize leaves by employing common signaling components of the LR and callus formation pathway, including *AtARF7/AtARF19* and LBDs. Therefore, we searched for maize orthologues of these pathway genes and identified one gene (*Zmarf27*) as the orthologue of both *AtARF7* and *AtARF19*, and two genes (*Zmlbd24* and *Zmlbd1*) as orthologues of *AtLBD16* (Fig. 5a; Berardini *et al*., 2015). We then examined their expression levels in *U. maydis‐*infected maize leaves compared to mock control by quantitative real‐time PCR (qRT‐PCR). We also included *rtcs*, and *ra2*, orthologues of *AtLBD29*, that is a closest homologue of class‐IB LBD (Fig. 5a) genes of *A. thaliana. Ra2* was previously found to be downregulated in the pentuple‐deletion‐mutant (*Δtips1‐5*)‐infected maize seedlings (Khan *et al*., 2024). Moreover, ra2‐like binding sites were enriched in differentially expressed genes from *Δtip6* mutant‐infected maize leaves (Huang *et al*., 2023). Strikingly, the expression levels of *Zmarf27*, *ra2*, and *Zmlbd1* were significantly and specifically induced in the leaves infected with *U. maydis* (Fig. 5b). At the same time, *rtcs* was consistently upregulated but not significantly different between *U. maydis*‐infected leaves compared with mock treatment in our assays. The transcript abundance of *Zmlbd24* was below detection limits in both *U. maydis‐infected* and mock leaves (data not shown). To further investigate the role of class II Tips, we examined the expression of these genes in the Tips pentuple mutant (*Δtips1‐5*; Bindics *et al*., 2022), in which three class II Tips (*Tip3*, *Tip4*, and *Tip5*) are absent, and observed a small but significant reduction in *ra2* and *Zmlbd1* expression.

**Fig. 6:**
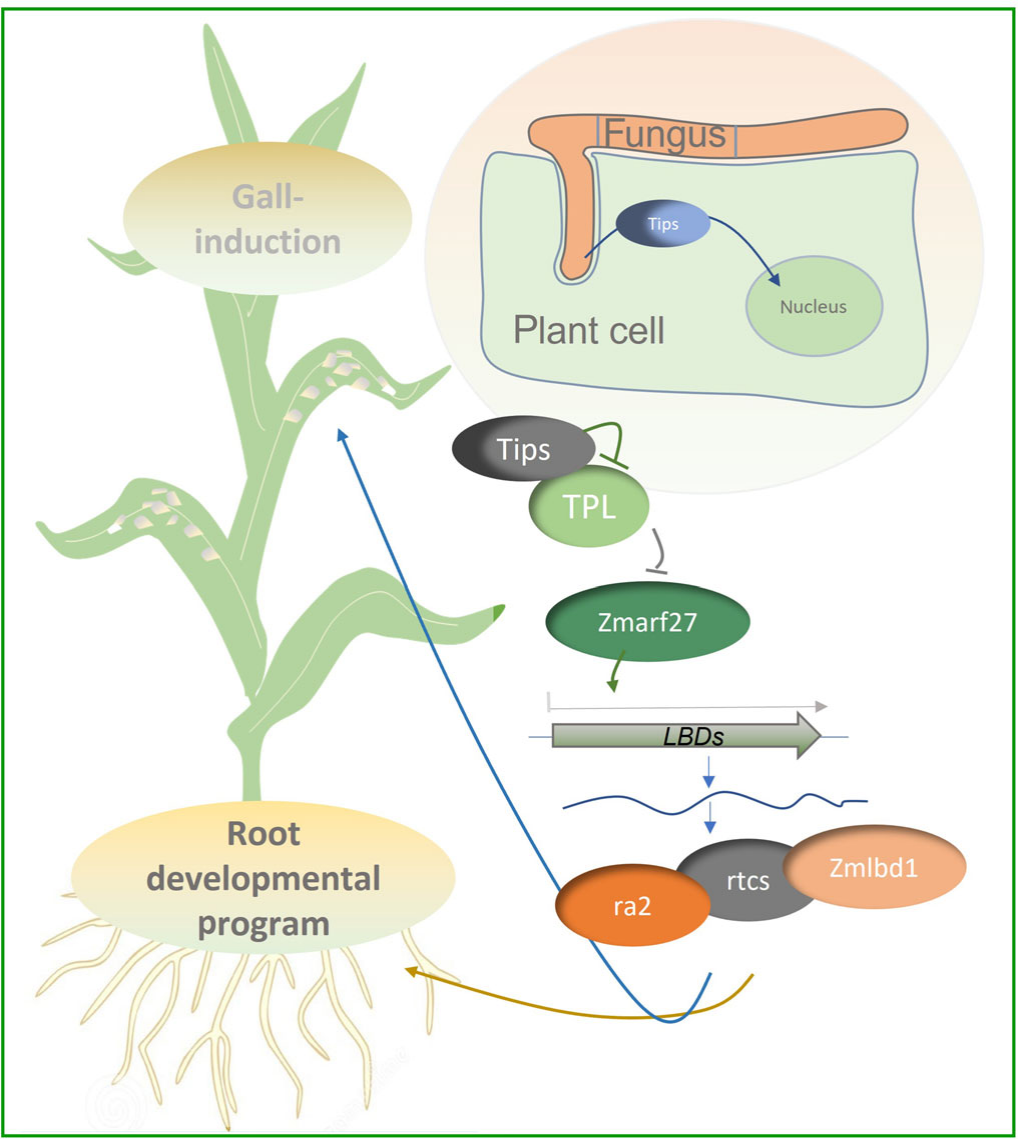
Working model for class II Topless (TPL)‐interacting protein effectors in maize shoot reprogramming. Class II TPL‐interacting protein (Tip3, Tip4, Tip5, Tip6, and Tip7) effectors translocate to the plant nucleus and interact with maize TPL proteins during *Ustilago maydis* biotrophy, leading to de‐repression of maize auxin response factor 27 (*Zmarf27*). Zmarf27 can then bind the promoters of specific LATERAL ORGAN BOUNDARIES DOMAIN transcription factors *ra2*, *rtcs*, and *Zmlbd1*, driving transcriptional reprogramming toward cellular dedifferentiation and gall formation in the leaves where they do not express under normal developmental conditions. Blunt ended arrow represents inhibition while a pointed arrow indicates upregulaton.

To elucidate the role of this pathway during *U. maydis* biotrophy, we next looked for the maize mutants of the respective LOB‐domain TFs and identified previously published mutants in *rtcs* and *ra2* genes. We tested the ability of the recessive *rtcs* and *ra2* loss‐of‐function mutants (Fig. S7) to form galls upon *U. maydis* infection. For this purpose, 9‐d‐old maize seedlings of segregating *rtcs‐1* and *ra2‐R* alleles were infected with the solopathogenic *U. maydis* strain SG200, and symptom scoring was performed 12 dpi. The results of this experiment show a slight but significant reduction in virulence in homozygous recessive mutants compared with the segregating population (Fig. 5c). Taken together, *U. maydis* induces the expression of *Zmarf27* and certain LBDs during its biotrophy in maize leaves, and single *rtcs* and *ra2* mutants show virulence defects, suggesting a role of this branch of auxin signaling during gall formation (Fig. 6). Moreover, the weaker virulence defects of recessive single mutants of LBD genes indicate their redundant roles in this process.

## Discussion

More than a century of *U*. *maydis* research has been in part driven also by the curiosity of how this fascinating biotrophic fungus can induce prominent galls on all aerial parts of its host plant maize (Küster, 1911). However, the underlying mechanisms remained elusive. Since the *U. maydis* genome has been published (Kamper *et al*., 2006), many effector genes have been placed into the context of gall formation based on studies of their deletion strains, which were impaired in pathogenicity and hence altered gall formation upon infection (Brefort *et al*., 2014; Redkar *et al*., 2015; Lanver *et al*., 2017; Djamei *et al*., 2023; Khan & Djamei, 2024). Nevertheless, it was unknown how many factors would be needed to induce galls *in planta* in the absence of fungal infection. Here, we provide significant insights through *in planta* by Tip effector overexpression, in which major phenotypic changes were observed and could be classified in two distinct classes. For the strongest representative of the class II Tip effectors, Tip 4, we provide genetic evidence that it derepresses *AtARF7* and *AtARF19* TFs, leading to activation of *LBD* genes involved in LR and callus formation. In contrast to the previously reported effectors, the class II Tip effectors act dominantly and independently of the presence of *U. maydis* infection, in which overexpression of a single effector is sufficient to induce an endogenously encoded cellular dedifferentiation and proliferation program. In the strongest phenotypes (Tip3, Tip4, and Tip6), this results in pluripotent callus formation at positions in which LRs would normally emerge, whereas Tip5 and Tip7 primarily alter LR frequency and length. All class II Tips also induce leaf chlorosis, although the severity varies among effectors. The phenotypic variation within this group suggests that, although all class II Tips act as negative regulators of TPL/TPR proteins, their activities display a degree of specificity. We further show an overlap in transcriptional programming between *U. maydis‐*induced gall formation in maize leaves and LR emergence. These results are completely novel in the *U. maydis–Zea mays* pathosystem and in line with recent findings in *A. thaliana* that demonstrate auxin‐induced callus formation occurs from the pericycle (or pericycle‐like cells) within multiple organs through a root development pathway, during which the ectopic activation of root meristematic genes is required for subsequent regeneration programs (Che *et al*., 2007; Sugimoto *et al*., 2010).

The discrepancy that *U. maydis* causes galls occurs solely on the aerial organs of its host plant maize, while class II Tips overexpressed in *A. thaliana* solely induce calli in the roots, which requires some consideration. One explanation could be that during *U. maydis* infection, the complex background manipulation by a whole effectome creates metabolic and transcriptional preconditioning, which cannot be recapitulated by expression of a single *U. maydis* effector. The transcriptional derepression of *AtLBD16* homologs (Fig. 5b) is therefore important but not sufficient to trigger callus formation in the shoot. Furthermore, this capacity appears to be restricted, as *U. maydis* induces cell division (hyperplastic leaf gall tissue) only in specific cell types, namely bundle sheath cells, rather than throughout the entire leaf (Matei *et al*., 2018). Nevertheless, class II Tips show clear effects also in the shoot of transgenic plants, that is chlorosis. This situation also occurs in *U. maydis* galls, which are sink tissues that show a loss of chloroplasts and turn pale yellow or red due to anthocyanin accumulation.

It has been demonstrated previously in *A. thaliana* that the formation of LRs, as well as the development of callus, both require several common regulatory components of the LR developmental pathways, that is *AtARF7*, *AtARF19*, *AtLBD16*, *AtLBD18*, *AtLBD29*, *and AtLBD33* (Sugimoto *et al*., 2010; Fan *et al*., 2012; Perianez‐Rodriguez *et al*., 2014). The formation of callus upon *Tip4* overexpression is almost abolished in the *arf7/arf19* double mutant background, placing *AtARF7* and *AtARF19* downstream of Tip4 and demonstrating their importance in Tip4‐induced callus formation in *A. thaliana*. The fact that there were still a few callus‐like structures appearing upon *Tip4* overexpression in the *arf7/arf19* mutant background could be due to the involvement of other ARFs, for example *AtARF5*, in this process (Vangheluwe & Beeckman, 2021). Leaves of *A. thaliana* plants expressing class II Tips turn chlorotic, and this phenomenon seems also to be dependent on *AtARF7* and *AtARF19* as the leaf phenotypes of Tip4 are widely rescued in the *arf7/arf19* mutant plants (Fig. 3o,p). We also observed this in western blots targeting class II Tips overexpression after already 5 d induction. This correlates with the yellowing of the leaf, and the Rubisco band in the ponceau‐staining of western blots is very faint (Fig. S8), indicating that not only Chl but also chloroplast function in total is negatively regulated by AtARF7 and AtARF19 activities. Consistent with this, GO‐enrichment analysis of the commonly downregulated genes between *U. maydis‐*infected maize leaves and LR initiation‐repressed genes revealed an overrepresentation of photosynthesis and chloroplast‐related components (Fig. 4g).

Gall formation is a widely occurring phenomenon caused by various pathogens such as gall wasps, bacteria such as Phytoplasmas, *Pantoea agglomerans*, *Pseudomonas savastanoi*, *Xanthomonas citri*, *Rhodococcus fascians*, the root‐knot and cyst nematodes, and certain rust fungi and smuts (Harris & Pitzschke, 2020). Gall formation might have several advantages for the colonization and proliferation in the host, including immune suppression (Navarrete *et al*., 2022) and efficient nutrient acquirement (Horst *et al*., 2010; Sosso *et al*., 2019). Furthermore, considering the biotrophic lifestyle of these pathogens, there is possibly also a reduction in interference with essential plant functionalities during massive proliferation in a separated tissue irrelevant for plant survival. We provide here evidence that *U. maydis* employs the postembryonic organogenesis pathway to induce galls. Unlike galls and giant cells formed by root‐knot nematodes, in which direct overexpression of *AtLBD16* is induced for callus formation, the class II Tip effectors of *U. maydis* induce callus formation upstream of AtARF7 and AtARF19 by distinct suppression of TPL functions. This highlights an example of convergent evolution of the pathway comprising TPL as a negative regulator of AtARF7 and AtARF19.

## Competing interests

None declared.

## Author contributions

MK and AD were involved in conceptualization, methodology. MK, NN and KS were involved in investigation. DW and PY were involved in transcriptomic data generation and analysis. MK was involved in project administration. MK, NN, CM, FH and AD were involved in resources. MK and AD were involved in original draft. AD and MK were involved in funding acquisition and supervision.

## Disclaimer

The New Phytologist Foundation remains neutral with regard to jurisdictional claims in maps and in any institutional affiliations.

## Supporting information


**Dataset S1.** Transcriptomic comparison of *U. maydis*‐infected maize leaves at 4 d post infection with gene expression in phloem‐pole pericycle cells from 6‐wk‐old maize, the site of lateral root initiation.
**Dataset S2**. List of 739 genes commonly induced in both datasets, functionally classified by Gene Ontology terms.
**Dataset S3**. List of 202 genes commonly downregulated in both datasets, functionally classified by Gene Ontology terms.

**Fig. S1** *A. thaliana* plants expressing Topless interacting protein effector 1 (Tip 1) show chlorophyll loss and inhibition of overall growth phenotypes.
**Fig. S2** *A. thaliana* plants expressing Topless interacting protein (Tip) effectors of class I showing chlorophyll loss and inhibition of overall growth phenotypes.
**Fig. S3** *A. thaliana* plants expressing Topless interacting protein (Tip) effectors of class II showing phenotypes of increased lateral roots/callus‐like structures and inhibition of root lengths.
**Fig. S4** The primary root lengths of *A. thaliana* seedlings expressing either pXVE:HAmCherry, or pXVE:HA‐mCherry‐Tip4.
**Fig. S5** The root explants of pXVE:HA‐mCherry‐Tip4 pre‐incubated with 10 μM estradiol for the induction of Tip4 expression were transferred to shoot‐inducing medium (SIM) to induce *de novo* shoot regeneration.
**Fig. S6** TOPLESS interacting protein (Tip)‐4 effector‐induced root callus formation requires AtLBD16 expression.
**Fig. S7** Characterization of ra2‐R and rtcs‐1 recessive mutants.
**Fig. S8** Arabidopsis. thaliana plants expressing Topless interacting protein effector 4 (Tip 4) show chlorophyll loss and absence of rubisco.
**Table S1** Summary of cell – death and other morphological phenotypes observed for Topless interacting protein (Tip) effectors of *Ustilago maydis* across different studies.
Please note: Wiley is not responsible for the content or functionality of any Supporting Information supplied by the authors. Any queries (other than missing material) should be directed to the *New Phytologist* Central Office.

## Data Availability

All data supporting the findings of this study is available within the article (Figure 4) and Supporting Information (Datasets S1‐S3). The raw RNA sequencing data that support the findings of this study are deposited in the sequence read archive (http://www.ncbi.nlm.nih.gov/sra) under accession no. PRJNA1366082 (https://www.ncbi.nlm.nih.gov/sra/?term=PRJNA1366082). Accession numbers: Zmarf27 (Zm00001eb373970), Zmlbd1 (Zm00001eb003910), Zmlbd24 (Zm00001eb191160), rtcs (Zm00001eb003920), ra2 (Zm00001eb123060), Tip1 (UMAG_11415), Tip2 (UMAG_02535), Tip3 (UMAG_02537), Tip4 (UMAG_02538) and Tip5 (UMAG_11417), Tip6 (UMAG_11060), Tip7 (UMAG_05300), Tip7 (UMAG_05308), Jsi1 (UMAG_001236), Nkd1 (UMAG_02299), AtARF7 (AT5G20730), AtARF19 (At1g19220), AtLBD16 (At2g42430), AtLBD25 (AT3G27650), AtLOB (At5g63090).
