# Supplementary material for: Pathogenic fungus Ustilago maydis exploits the lateral root regulators to induce pluripotency in maize shoots
Source: New Phytol. 2025 Dec 26;249(6):2974–85. doi: 10.1111/nph.70843 (PMC12917459; doi:10.1111/nph.70843)
Supplement: Supplementary file 2 — Fig. S1 A. thaliana plants expressing Topless interacting protein effector 1 (Tip 1) show chlorophyll loss and inhibition of overall growth phenotypes. Fig. S2 A. thaliana plants expressing Topless interacting protein (Tip) effectors of class I showing chlorophyll loss and inhibition of overall growth phenotypes. Fig. S3 A. thaliana plants expressing Topless interacting protein (Tip) effectors of class II showing phenotypes of increased lateral roots/callus‐like structures and inhibition of root lengths. Fig. S4 The primary root lengths of A. thaliana seedlings expressing either pXVE:HAmCherry, or pXVE:HA‐mCherry‐Tip4. Fig. S5 The root explants of pXVE:HA‐mCherry‐Tip4 pre‐incubated with 10 μM estradiol for the induction of Tip4 expression were transferred to shoot‐inducing medium (SIM) to induce de novo shoot regeneration. Fig. S6 TOPLESS interacting protein (Tip)‐4 effector‐induced root callus formation requires AtLBD16 expression. Fig. S7 Characterization of ra2‐R and rtcs‐1 recessive mutants. Fig. S8 Arabidopsis. thaliana plants expressing Topless interacting protein effector 4 (Tip 4) show chlorophyll loss and absence of rubisco. Table S1 Summary of cell – death and other morphological phenotypes observed for Topless interacting protein (Tip) effectors of Ustilago maydis across different studies. Please note: Wiley is not responsible for the content or functionality of any Supporting Information supplied by the authors. Any queries (other than missing material) should be directed to the New Phytologist Central Office. [file NPH-249-2974-s002.pdf]

New Phytologist Supporting Information

Pathogenic fungus *Ustilago maydis* exploits the lateral root regulators to induce pluripotency in maize shoots

Mamoona Khan, Nithya Nagarajan, Kathrin Schneewolf, Caroline Marcon, Danning Wang, Frank Hochholdinger, Peng Yu, and Armin Djamei

Article acceptance date: 23 November 2025

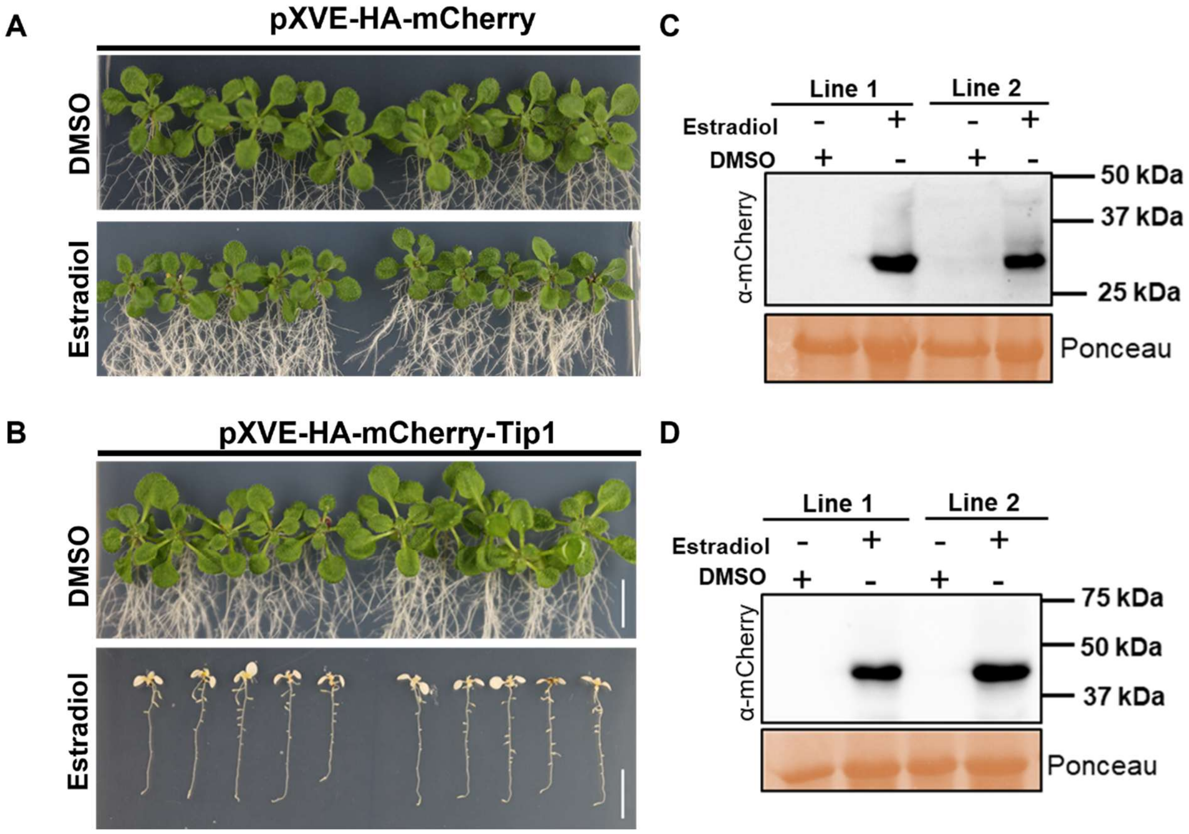

Fig. S1 ***Arabidopsis thaliana*** **plants expressing Topless interacting protein effector 1 (Tip 1) show chlorophyll loss and inhibition of overall growth phenotypes.** Seven-day-old ½ MS agar-grown seedlings expressing (A) *pXVE:HA-mCherry* (B) *pXVE: HA-mCherry-Tip1* were moved to either DMSO or 10µM estradiol containing ½ MS agar plates, and images were taken at 10 days after the transfer. In each panel, right and left are two independent lines, scale bar = 1cm (C, D). Western blot analysis of total protein extracts of 8-day-old plants treated with either DMSO or 20 µM estradiol for 2 hours. Membranes were incubated with α-mCherry antibody. Ponceau staining shows a loading control.

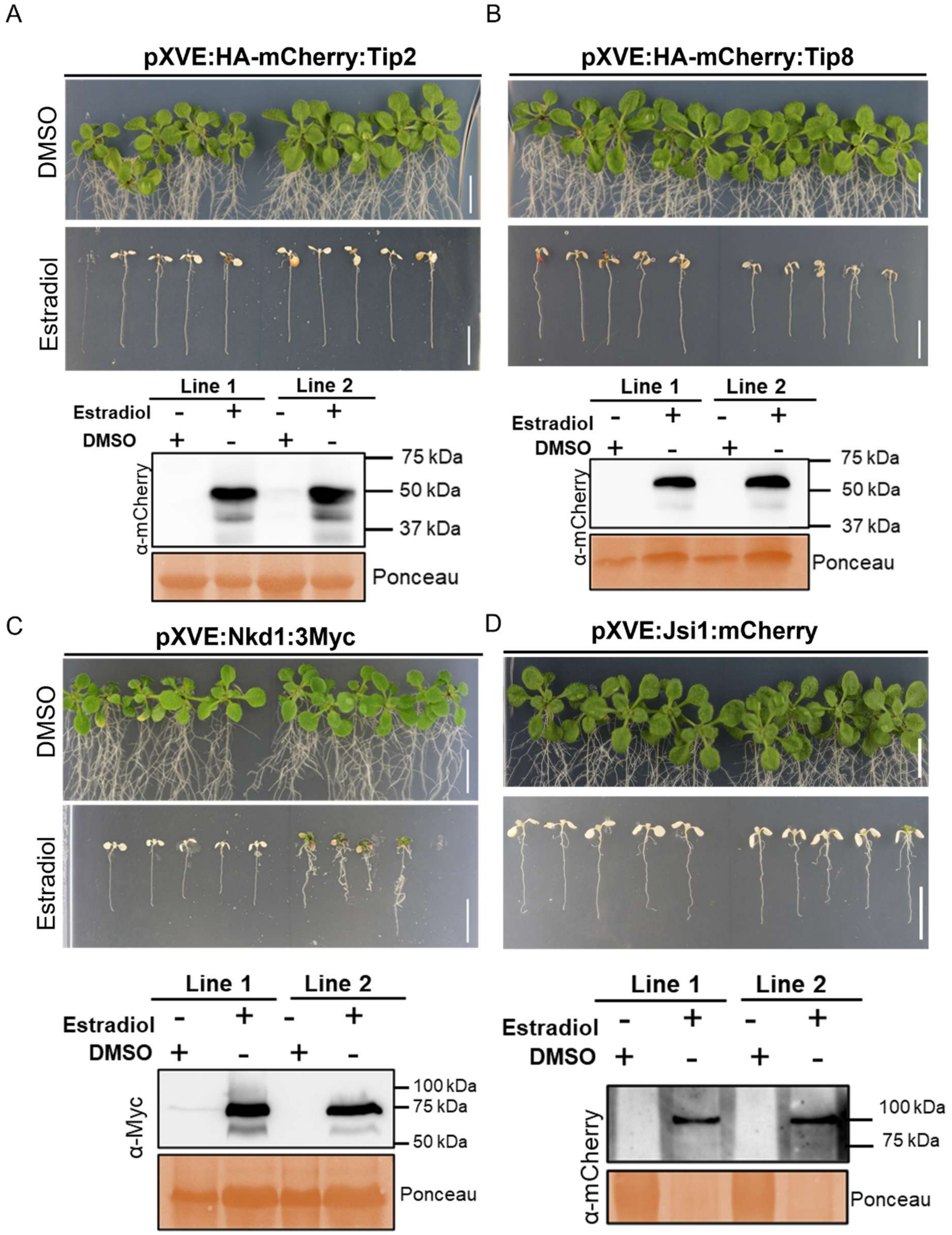

A
pXVE:HA-mCherry:Tip2
DMSO
Estradiol
Line 1
Line 2
Estradiol - + - +
DMSO + - + -
α-mCherry
75 kDa
50 kDa
37 kDa
Ponceau
B
pXVE:HA-mCherry:Tip8
DMSO
Estradiol
Line 1
Line 2
Estradiol - + - +
DMSO + - + -
α-mCherry
75 kDa
50 kDa
37 kDa
Ponceau
C
pXVE:Nkd1:3Myc
DMSO
Estradiol
Line 1
Line 2
Estradiol - + - +
DMSO + - + -
α-Myc
100 kDa
75 kDa
50 kDa
Ponceau
D
pXVE:Jsi1:mCherry
Line 1
Line 2
Estradiol - + - +
DMSO + - + -
α-mCherry
100 kDa
75 kDa
Ponceau

Fig. S2 ***Arabidopsis thaliana*** **plants expressing Topless interacting protein (Tip) effectors of class I showing chlorophyll loss and inhibition of overall growth phenotypes.** Seven-day-old ½ MS agar-grown seedlings expressing (A) *pXVE:HA- mCherry-Tip2* (B) *pXVE:HA-mCherry-Tip2* (C) *pXVE:Nkd1-3myc* and (D) *pXVE:Jsi-mCherry* were moved to either DMSO or 10µM estradiol containing ½ MS agar plates and images were taken at 10 days after the transfer. In each panel, right and left are two independent lines, scale bar = 1cm. Lower panels show western blot analysis of total protein extracts of 8-day-old plants treated with DMSO or 20 µM estradiol for 2 hours. Membranes were incubated with α-mCherry antibody. Ponceau staining shows a loading control.

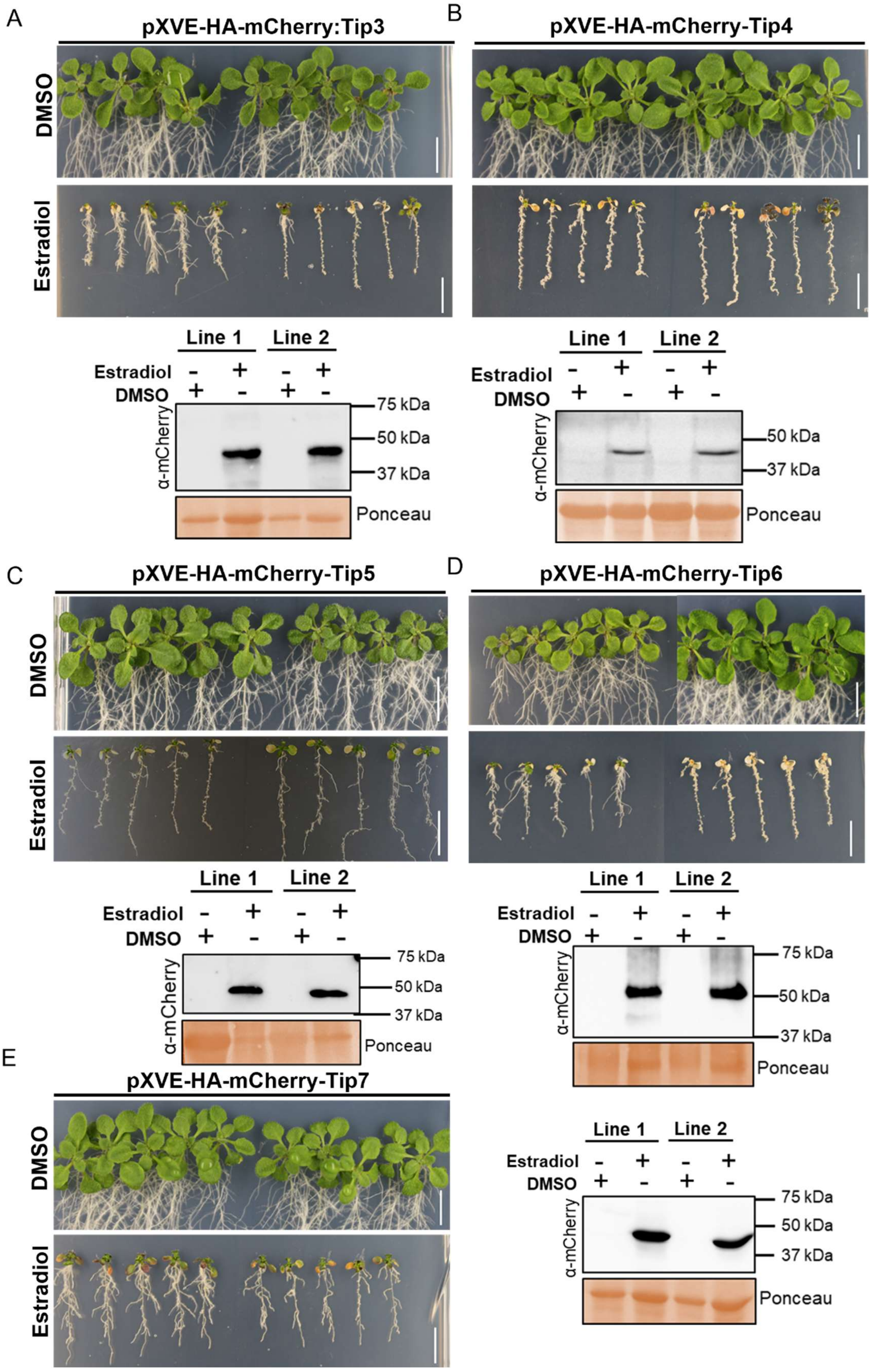

A
pXVE-HA-mCherry:Tip3
DMSO
Estradiol
Line 1
Line 2
Estradiol - + - +
DMSO + - + -
α-mCherry
75 kDa
50 kDa
37 kDa
Ponceau
B
pXVE-HA-mCherry-Tip4
DMSO
Estradiol
Line 1
Line 2
Estradiol - + - +
DMSO + - + -
α-mCherry
50 kDa
37 kDa
Ponceau
C
pXVE-HA-mCherry-Tip5
DMSO
Estradiol
Line 1
Line 2
Estradiol - + - +
DMSO + - + -
α-mCherry
75 kDa
50 kDa
37 kDa
Ponceau
D
pXVE-HA-mCherry-Tip6
Line 1
Line 2
Estradiol - + - +
DMSO + - + -
α-mCherry
75 kDa
50 kDa
37 kDa
Ponceau
E
pXVE-HA-mCherry-Tip7
DMSO
Estradiol
Line 1
Line 2
Estradiol - + - +
DMSO + - + -
α-mCherry
75 kDa
50 kDa
37 kDa
Ponceau

Fig. S3 ***Arabidopsis thaliana*** **plants expressing Topless interacting protein (Tip) effectors of class II showing phenotypes of increased lateral roots / callus-like structures and inhibition of root lengths.** Seven-day-old ½ MS agar-grown seedlings expressing (A) *pXVE:HA- mCherry-Tip3* (B) *pXVE:HA-mCherry-Tip4* (C) *pXVE:HA-mCherry-Tip5* (D) *pXVE:HA-mCherry-Tip6* and (E) *pXVE:HA-mCherry-Tip7* were moved to either DMSO or 10µM estradiol containing ½ MS agar plates, and images were taken at 10 days after the transfer. In each panel right and left are two independent lines, scale bar = 1cm. Lower panels show western blot analysis of total protein extracts from 8-day-old plants treated with either DMSO or 20 µM estradiol for 2 hours. Membranes were incubated with α-mCherry antibody. Ponceau staining shows a loading control.

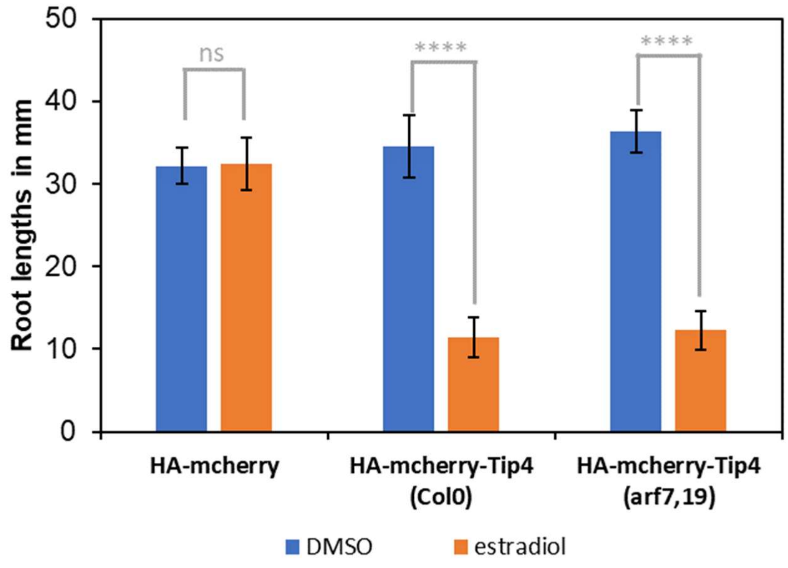

Fig. S4 The primary root lengths of *A. thaliana* seedlings expressing either *pXVE:HA-mCherry*, or *pXVE:HA-mCherry-Tip4.* Seven-day-old *A. thaliana* seedlings expressing *pXVE:HA-mCherry*, *pXVE:HA-mCherry-Tip4* in Columbia (Col0) background, or *pXVE: HA-mCherry-Tip4* in *arf7arf19* background were transferred to ½ MS agar plates containing either DMSO or 10 µM estradiol, and their root lengths were measured at 8 days after transfer (DAT). Significant differences between DMSO and estradiol-treated plants were evaluated by Student's t-test analysis (**** = $p < 0.001$, ns = not significant). N= 10, and the experiment was repeated at least three times.

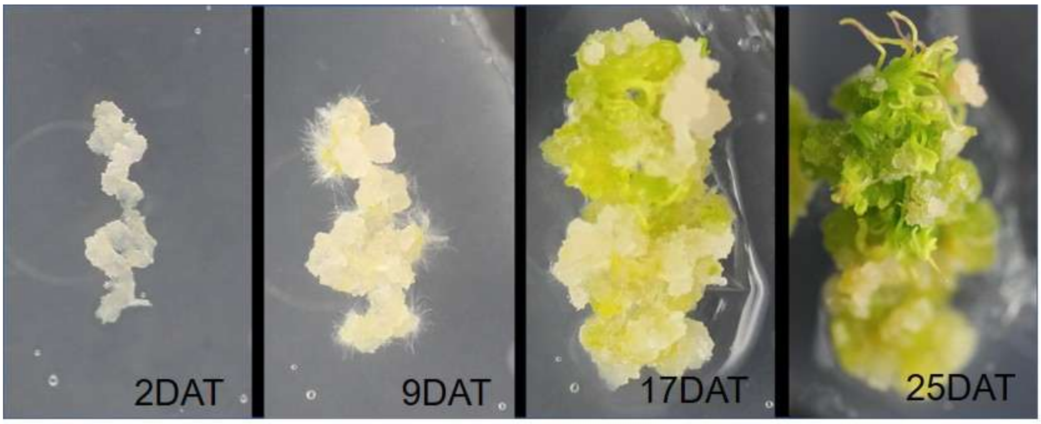

Fig. S5 The root explants of *pXVE:HA-mCherry-Tip4* pre-incubated with 10μM estradiol for the induction of Tip4 expression were transferred to shoot-inducing medium (SIM) to induce de novo shoot regeneration. The SIM plates were incubated under long-day conditions, and images were taken 2 days after the transfer (DAT), 9 DAT, 17 DAT, and 25 DAT to SIM.

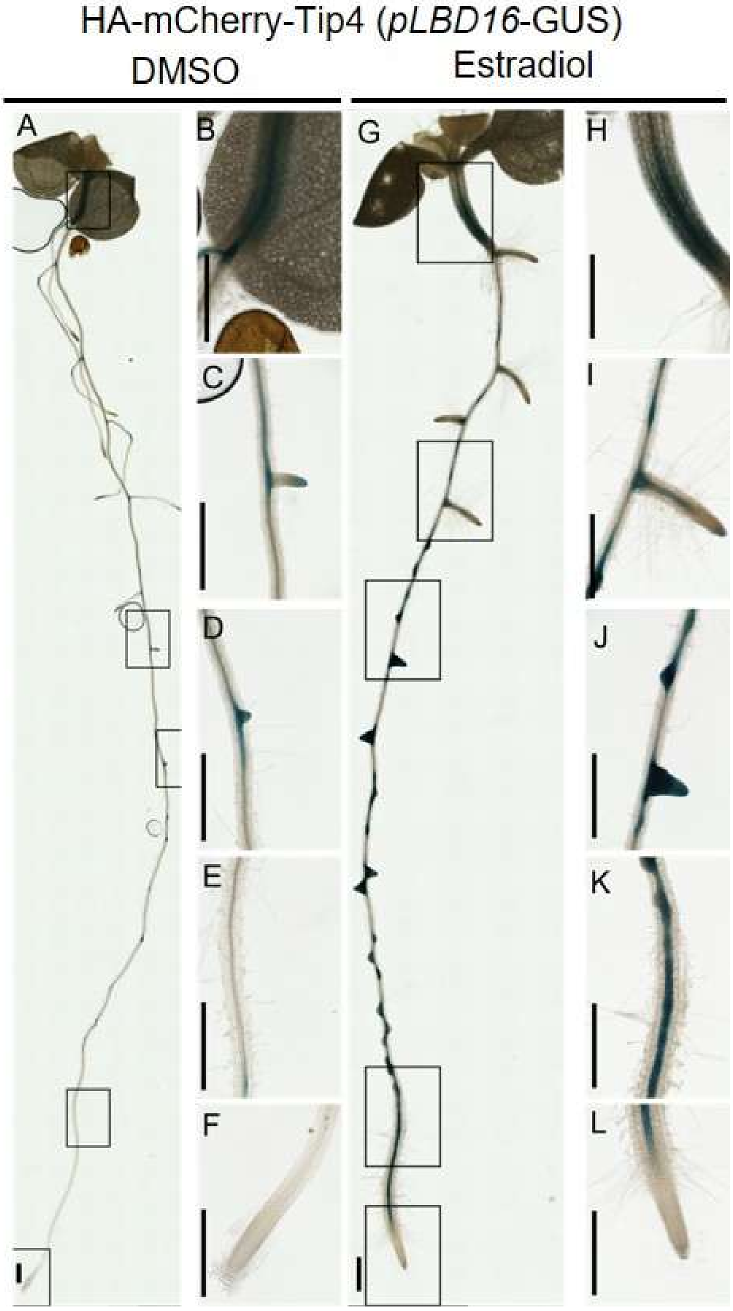

Fig. S6 TOPLESS interacting protein (Tip)-4 effector-induced root callus formation requires *AtLBD16* expression. (A–L) Digital microscopy images of seedlings expressing *pLBD16:GUS* in a *pXVE:HA-mCherry-Tip4* background. Seven-day-old seedlings grown on ½ MS agar were transferred to either DMSO-containing medium (A- F) or 10 μM estradiol (G-L) for 4 days before β-glucuronidase (GUS) staining, scale bar = 500 μm.

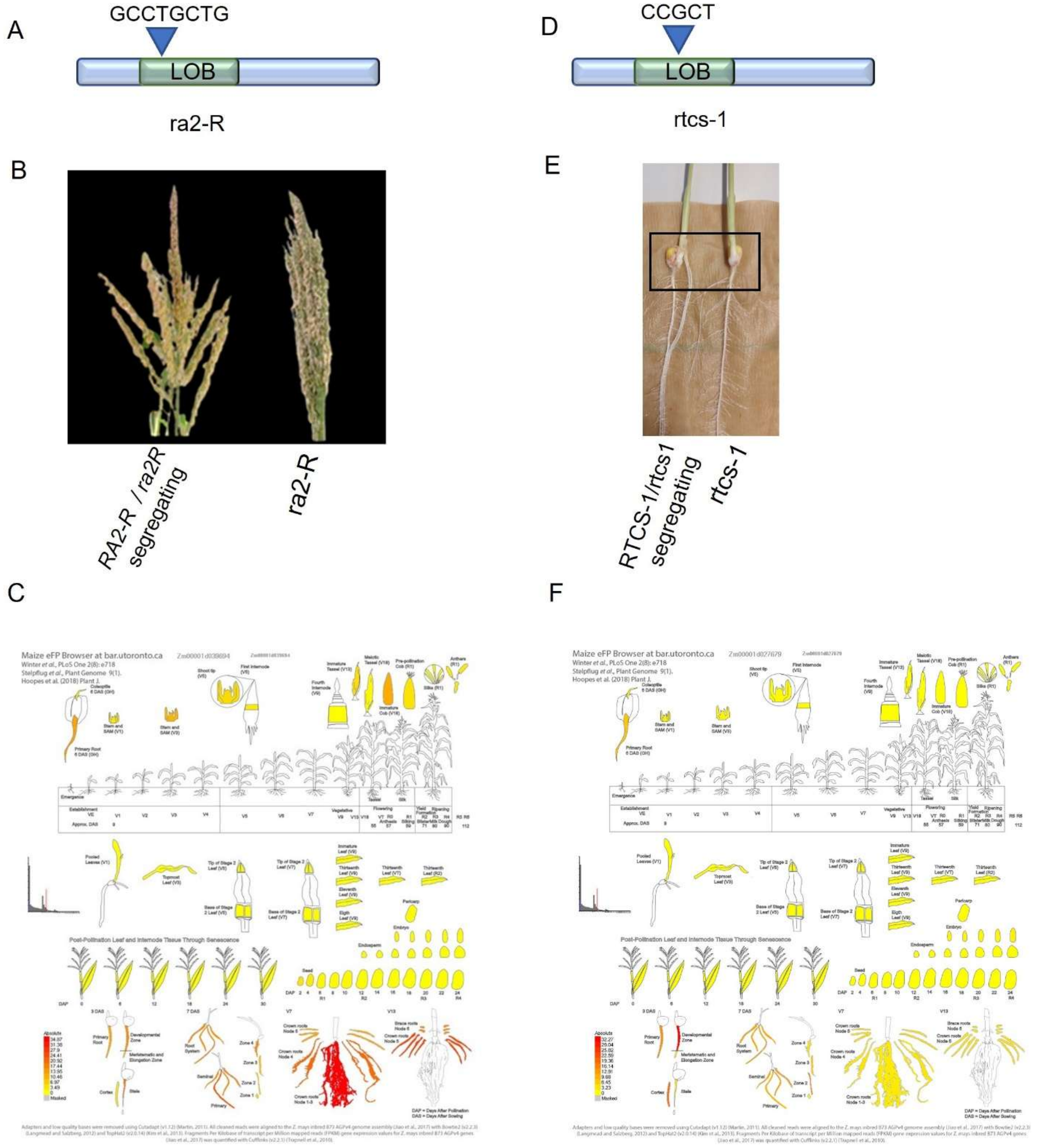

Fig. S7 Characterization of *ra2-R* and *rtcs-1* recessive mutants. (A) The *ra2-R* allele has an eight-base-pair (bp) insertion in the LATERAL ORGAN BOUNDARIES (LOB)- DOMAIN of *Ramosa2* (*RA2*) that introduces a stop codon within the LOB domain. (B) The tassel phenotype of the *ra2-R* mutant compared to the segregating control. (C) Tissue-specific expression of *RA2*

(Zm00001d039694) according to (Hoopes *et al.*, 2019; Woodhouse *et al.*, 2021). (D) The *rtcs-1* allele has a five-base-pair insertion in the LATERAL ORGAN BOUNDARIES (LOB)- DOMAIN of *RTCS* that introduces a stop codon 227 bp downstream of the putative ATG start codon. (E) The phenotype of 8-day-old *rtcs-1* mutant; it does not form shoot-borne roots compared to the segregating control. (F) Tissue-specific expression of *RTCS* (Zm00001d027679) according to (Winter *et al.*, 2007; Hoopes *et al.*, 2019; Woodhouse *et al.*, 2021).

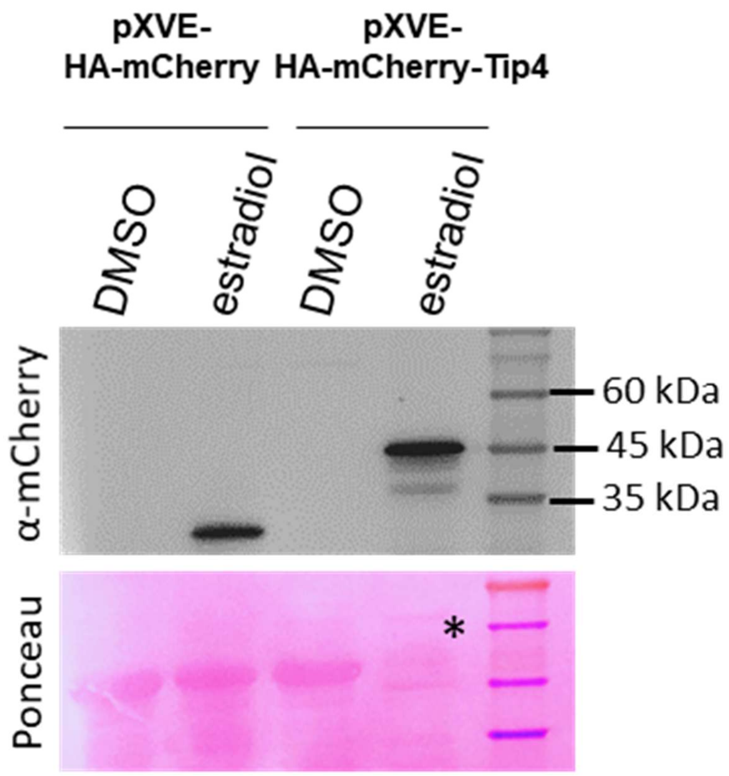

Fig. S8 ***Arabidopsis thaliana*** **plants expressing Topless interacting protein effector 4 (Tip 4) show chlorophyll loss and absence of rubisco.** Seven-day-old ½ MS agar-grown seedlings expressing either *pXVE:HA-mCherry* or *pXVE: HA-mCherry-Tip4* were moved to either DMSO or 10µM estradiol containing ½ MS agar plates, and a western blot was performed 10 days after the transfer. 100 mg of plant material was used for protein extraction. Membrane was incubated with α-mCherry antibody. Ponceau staining of the membrane shows the absence of Rubisco when Tip4 was induced, marked by asterisk.

**Name of Tip**	**Cell death inducer in *Nicotiana benthamiana***	**Lateral root inducer in *Arabidopsis thaliana***	**Classified into a phenotypic class in this study**
Tip1 (umag_11415)	Yes (strong), (Bindics *et al.*, 2022)	No, (this study)	Class I
Tip2 (umag_02535)	yes (strong), (Bindics *et al.*, 2022)	No, (this study)	Class I
Tip3 (umag_02537)	No, (Bindics *et al.*, 2022)	Yes, (this study)	Class II
Tip4 (umag_02538)	No, (Bindics *et al.*, 2022)	strong inducer, (this study)	Class II
Tip5 (umag_11416)	No, (Bindics *et al.*, 2022)	weak inducer, (this study)	Class II
Tip6 (umag _11060)	No, (Khan *et al.*, 2024)	weak inducer, (this study)	Class II
Tip7 (umag_05300)	No, (Khan *et al.*, 2024)	weak inducer, (this study)	Class II
Tip8 (umag_05308)	Yes, (Khan *et al.*, 2024)	No, (this study)	Class I
Nkd1 (umag_02299)	Yes (strong), Navarrete et al 2022	No, (this study)	Class I
Jsi1 (umag_01236)	Yes (strong), (Darino *et al.*, 2021)	No, (this study)	Class I

Table S1 Summary of cell - death and other morphological phenotypes observed for Topless-interacting protein (Tip) effectors of *Ustilago maydis* across different studies.

Supplementary References

Bindics J, Khan M, Uhse S, Kogelmann B, Baggely L, Reumann D, Ingole KD, Stirnberg A, Rybecky A, Darino M, et al. 2022. Many ways to TOPLESS - manipulation of plant auxin signalling by a cluster of fungal effectors. *New Phytol* **236**(4): 1455-1470.

Darino M, Chia KS, Marques J, Aleksza D, Soto-Jimenez LM, Saado I, Uhse S, Borg M, Betz R, Bindics J, et al. 2021. Ustilago maydis effector Jsi1 interacts with Topless corepressor, hijacking plant jasmonate/ethylene signaling. *New Phytol* **229**(6): 3393-3407.

Hoopes GM, Hamilton JP, Wood JC, Esteban E, Pasha A, Vaillancourt B, Provart NJ, Buell CR. 2019. An updated gene atlas for maize reveals organ-specific and stress-induced genes. *Plant J* **97**(6): 1154-1167.

Khan M, Uhse S, Bindics J, Kogelmann B, Nagarajan N, Tabassum R, Ingole KD, Djamei A. 2024. Tip of the iceberg? Three novel TOPLESS-interacting effectors of the gall-inducing fungus Ustilago maydis. *New Phytol*.

Winter D, Vinegar B, Nahal H, Ammar R, Wilson GV, Provart NJ. 2007. An "Electronic Fluorescent Pictograph" browser for exploring and analyzing large-scale biological data sets. *PLoS One* **2**(8): e718.

Woodhouse MR, Cannon EK, Portwood JL, 2nd, Harper LC, Gardiner JM, Schaeffer ML, Andorf CM. 2021. A pan-genomic approach to genome databases using maize as a model system. *BMC Plant Biol* **21**(1): 385.
